# Influence of age, gender, and radiographic features on the deltoid splitting approach for surgical osteosynthesis in displaced proximal humerus fractures: a comparison study

**DOI:** 10.1186/s12891-017-1840-6

**Published:** 2017-11-28

**Authors:** Alvin Chao-Yu Chen, Chih-Hao Chiu, Kuo-Yau Hsu, Yi-Sheng Chan

**Affiliations:** Bone and Joint Research Center, Department of Orthopaedic Surgery, Chang Gung Memorial Hospital-Linkou and University College of Medicine, 333, 5th, Fu-Hsin St., Kweishan Dist., Taoyuan, 333 Taiwan, Republic of China

**Keywords:** Proximal humerus fracture, Neer classification, Osteosynthesis, Deltoid splitting, Locking plate

## Abstract

**Background:**

The deltoid splitting approach has recently been widely adopted to facilitate less invasive procedures for proximal humerus fractures. However, there are still concerns regarding its effectiveness in aging people and in cases involving complex fractures. This study was conducted to evaluate the efficacy of a proximal humeral locking plate using the anterolateral deltoid splitting approach and to specifically examine the effect of patient age, gender, and fracture pattern on surgical outcomes.

**Methods:**

Forty-two cases of proximal humeral fractures treated using the deltoid splitting approach and locking plate fixation were reviewed. Outcome differences were evaluated in terms of age, gender distribution, and radiographic analysis based on the Neer Classification. The influence of the surgical approach was further investigated by age-matched paired analysis after subdividing patients into two age groups (younger than 60 years vs. older than 60 years; *N* = 21, in each group).

**Results:**

In total, 41 patients (98%) demonstrated fracture union. The average Constant score was 80.4. No significant differences were found between patients younger than 60 years, and the older patients. Higher mean scores were found in men than in women (*p* = 0.448) and in simple fractures than in complex fractures (*p* = 0.454), without any significant differences. Better postoperative functional outcomes were observed when the humeral head–neck angle was greater than 105°, with a significant difference (*p* = 0.000). Surgical complications were found in 16 patients (38%) without significant difference between two age groups (*p* = 0.268). The most common complication was screw penetration.

**Conclusions:**

Anterolateral deltoid splitting using locking plate fixation provided a feasible alternative for surgery of proximal humerus fractures in different age groups and yielded comparable outcomes when the neck-shaft angle was properly restored. Surgeons must be cautious regarding potential complications, especially with screw penetration when using the locking plate through a less invasive approach.

**Trial registration:**

ISRCTN75494532. Trial Date: 2017/01/31.

## Background

Proximal humeral fractures are among the most frequent fractures associated with osteoporosis, and may represent a major cause of functional disability [1, 2]. Unfortunately, the treatment of these fractures remains controversial, and surgical complications are common [3, 4]. The introduction of locked plating offers a novel biomechanical approach in stabilizing these fractures [5, 6]. Surgical approach using anterolateral deltoid splitting is a less invasive technique [7–9] in which the axially nerve is carefully located and protected [10], while avoiding potential disadvantages of extensive dissection and muscle retraction in the traditional deltopectoral approach [1, 11, 12]. However, some reports have expressed concerns regarding potential construct failures and inferior surgical outcomes in patients with more complex fractures and in an aging population [13, 14]. The present study aimed to evaluate the influence of the surgical approach for management of displaced proximal humerus fractures and to specifically examine the impact of patient age and fracture pattern on the outcomes.

## Methods

We retrospectively evaluated cases of unilateral proximal humerus fractures that were treated surgically with the Locking Compression Plate (LCP), as per the Neer classification criteria at our institute between 2009 and 2011. Institutional review board approval (No. 201700826B0) was obtained to perform a review of patients’ records and radiographs; informed consent was obtained from 132 patients with displaced proximal humerus fractures. All these cases of proximal humerus fractures either met the indications for operative treatment outlined by Neer [15] or were considered unstable when tested for passive motion with an image intensifier. Patients with pathological fractures, head split fractures, open fractures, fractures with primary neurovascular damage, multiple fractures, and cases lost to follow-up were excluded from the study. Forty-two patients, who underwent anterolateral deltoid splitting surgery, were selected for a retrospective matched pairs analysis according to their age (younger than 60 years vs. older than 60 years), gender, and fracture type (Table 1) with a minimum follow-up of 24 months. One single surgeon performed all the surgeries. Preoperative assessment showed 10 patients in younger group and 15 patients in older group had chronic diseases; most common was hypertension (3 and 10 in younger and older group respectively) and diabetes mellitus (3 and 6 in younger and old group respectively).

**Table 1:** Demographic data of patients

Characteristics	Age < 60-years-old	Age > 60-years-old	*P* value
Mean age	50	66	
Gender			0.333
Women	14 (67%)	15 (71%)	
Men	7 (33%)	6 (29%)	
Neer classification			0.374
Two-part	9 (43%)	10 (48%)	
Three- & four-part	12 (57%)	11 (52%)	
Time to surgery (days)	3	2.62	0.262

**Fig. 1:**
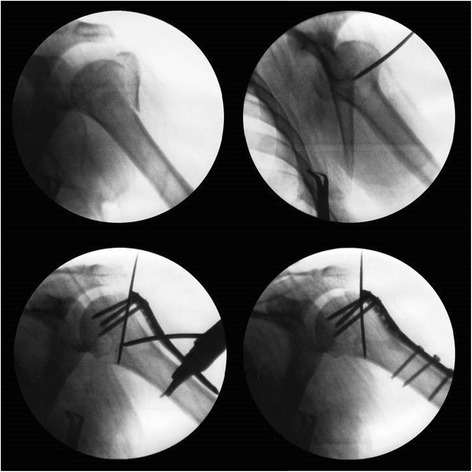
The fracture pattern is assessed by using a C-arm image intensifier (top left). Reduction is achieved by elevating the humeral head fragment (top right). Provisional wire fixation is applied, followed by insertion of a locking plate and proximal screws through a deltoid splitting approach (bottom left). Distal screws are applied through another skin incision (bottom right)

All procedures were performed in the beach-chair position on a radiolucent Table. A skin incision was made from the anterolateral lip of the acromion and was extended approximately 3 to 4 cm distally. The subcutaneous tissue layer was then bluntly dissected to identify the avascular raphe separating the anterior and middle head of the deltoid. Deltoid muscle splitting was limited to within 3 cm of the acromial end. The axial nerve was palpated and protected, but not dissected. The greater tuberosity was reduced by pulling forward the stay sutures on the bony fragment. Provisional Kirschner wire fixation was used to temporarily secure fracture fragments. A PHILOS plate (Synthes, Switzerland) was applied using the technique of minimally invasive plate osteosynthesis (MIPO) [16]. The plate was inserted along the anterolateral cortex of the humeral shaft while avoiding touching or hinging on the axial nerve branch. A second skin incision was made about 5 to 7 cm distal to the first incision to facilitate plate positioning along the proximal humerus shaft. Fracture reduction and plate location was then confirmed as adequate by using an image intensifier before definite fixation (Fig. 1). After placing the plate securely, four locking screws were inserted into the humeral head through the first incision and three screws for the shaft were applied through the second skin incision. For better visualization of screw holes and easier screw placement on the humeral head, the humerus was elevated to a 30-degree abduction position to relieve muscle tension and to avoid excessive retraction of the deltoid muscle. The Kirschner wires were then removed, and tension band fixation was applied to secure the rotator cuff using the premeditated Ethibond sutures. The surgical wound was closed with 3-O Vicryl sutures.

Functional evaluation was done using Constant Scores [17], which were further graded as excellent (90–100 points), good (80–89 points), fair (70–79 points), or poor (less than 70 points). In a secondary comparison, patients were divided into two age groups: a group of less than 60-year-old patients (*n* = 21) and a group of more than 60-year-old patients (*n* = 21). Constant scores at 1-year follow up were further compared between the two age groups in terms of distribution by gender, fracture patterns, and complication rates to determine the differences in outcome with index surgery.

### Statistical analysis

Data analyses were performed with the SPSS 18 statistical software for Windows. For normally distributed data (patient age and time between fracture and fixation) an independent sample t-test was used. For data that were not normally distributed (the Constant shoulder score), the Mann–Whitney rank sum test was used. For categorical data (gender and fracture pattern), a chi-square test was used. A *p*-value of <0.05 was considered statistically significant.

## Results

Time to surgery averaged at 2.81 ± 1.89 days; it was 3 ± 2.26 days in the younger group (age younger than 60 years) and 2.62 ± 1.47 in the aged group (over 60 years old) respectively. The difference was not significant (*p* = 0.262). There were 11 men and 31 women; the average age was 58.33 years (range, 36 to 77 years). The difference of gender distribution in the younger and older groups was not significant (*p* = 0.448; Table 2.). Nineteen cases were of two-part fractures (45%); among them, 4 fractures were long oblique fractures (10%) that extended to the metaphyseal and the proximal diaphyseal regions. Fourteen cases were of three-part fractures (33%) and 9 were of four-part fractures (21%). In total, 23 out of 42 cases involved complex (Neer 3- and 4-part) fractures with 12 and 11 such fractures seen in the younger and older groups, respectively; the difference was not significant (*p* = 0.374).

**Table 2:** Comparison of functional and radiographic outcomes

Patients	No. of cases	Constant score	*P* value
All cases	42	80.4	
Age			0.054
< 60-years-old	21	83.19	
> 60-years-old	21	77.19	
Gender			0.448
Men	13	80.69	
Women	29	80.28	
Fracture pattern†			0.454
Simple	19	80.63	
Complex	23	80.22	
Neck shaft angle			0.000*
> 105∘	21	86.24	
< 105∘	21	74.57	

† Neer classification: simple, two-part fracture; three- four-part fracture

*Statistically significant

**Fig. 2:**
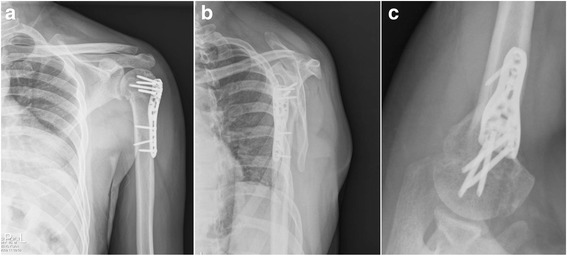
Postoperative radiographs at three months post-surgery, showing osseous union. **a** Anteroposterior projection. **b** Lateral projection. **c** Axial projection

Radiographic follow-up revealed complete osseous union within 6 months (Fig. 2) in 41 fractures (98%). The mean overall Constant score was 80.4 ± 10.66. Functional grading according to Constant score was good or excellent in 25 of 42 patients (60%). In analysis of age-matched pairs, the average Constant scores were 83.19 ± 7.61 for patients younger than 60 years (*n* = 21) and 77.19 ± 12.59 for patients older than 60 years (*n* = 21); the difference was not statistically significant (*p* = 0.054). Regarding gender differences, the average Constant score for female patients (*n* = 26) was 80.28 ± 7.37 and for male patients (*n* = 13) was 80.69 ± 11.74; the difference was not statistically significant (*p* = 0.448).

**Fig. 3:**
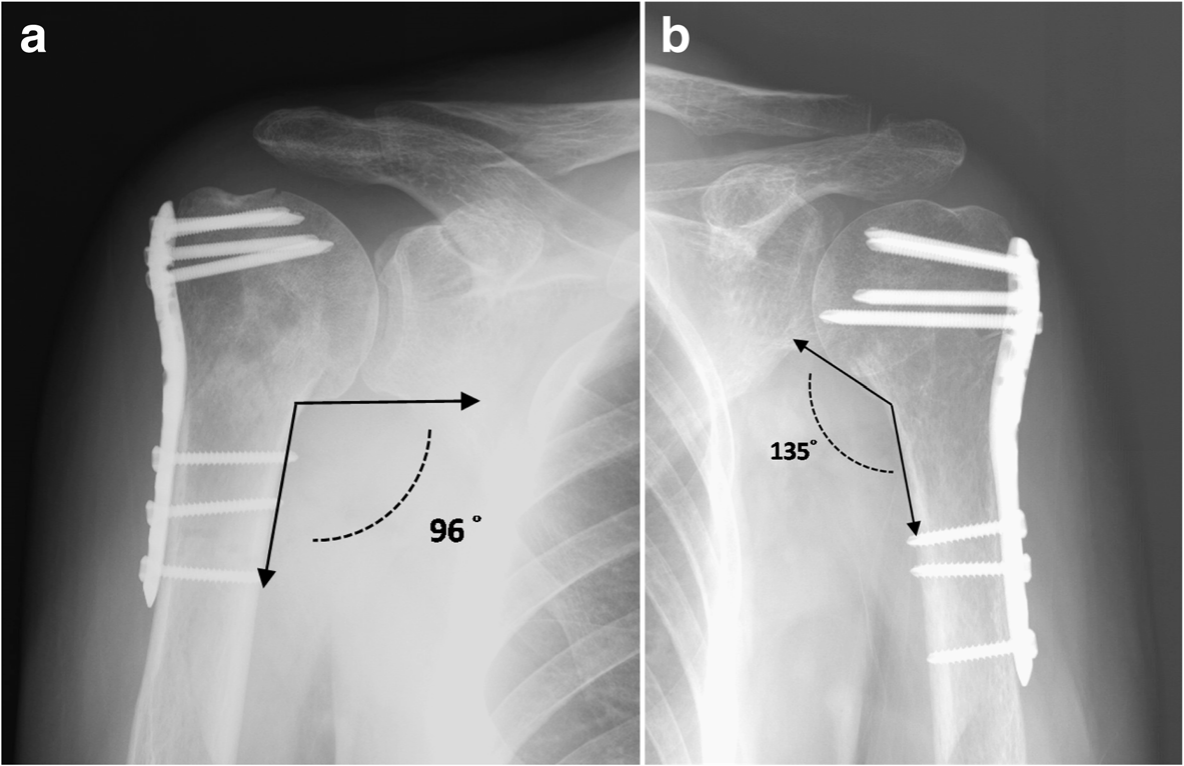
A comparison of the postoperative neck-shaft angles. **a** Varus alignment with an angle of 96∘. **b** Neutral position with an angle of 135∘

Based on Neer fracture classification, the average Constant score was 80.63 ± 13.87 for simple fractures (2-part; *n* = 19) and 80.22 ± 7.37 for complex fractures (three- and four-part; *n* = 23). The difference was not significant (*p* = 0.454). One of the 4 patients had long, spiral two-part fractures, which remained ununited at the 2-year follow-up and showed a poor result; the other 3 showed good or excellent results. On reviewing postoperative radiographs (Fig. 3), patients with humeral head–neck angles greater than 105° (*n* = 21, mean angle 119°) tended to have better outcomes (mean Constant score = 86.24) than those with angles lesser than 105° (*n* = 21, mean angle 97.19°, mean Constant score = 74.57). The difference was statistically significant (*p* = 0.000).

Immediate postoperative complications or neurovascular injuries were not found. No infections or wounds were observed. Complications observed within 2 years included screw penetration, avascular necrosis, and nonunion. Screw protrusion was observed in 12 patients (29%) with 5 and 7 in young and older groups respectively. Four patients (12%) developed subsequent necrosis of the humeral head; 2 were in each group. One patient had a second surgery with hemiarthroplasty 6 months after the osteosynthesis surgery. One patient (2%) with a two-part fracture had an accidental fall 6 weeks after surgery; radiographs revealed a secondary displacement of the humeral head. She underwent hemiarthroplasty; however, the patient had another accidental fall 1 month later, which caused a periprosthetic humeral fracture and a proximal ulnar fracture. The fracture remained unhealed at the 2-year follow-up. Both of the patients with hemiarthroplasty were in older group. In total, out of 16 patients (38%) who had surgical complications, 7 (33%) were in the younger group and the other 9 (43%) were in the older group. The incidence of complications was not statistically different between the two age groups (*p* = 0.268).

## Discussion

In an aging society, osteoporosis-related fractures and their comorbidities have increasingly become major medical concerns among elderly people [18]. The ultimate aim in treating these fractures in a geriatric population is to achieve a rapid bony union and allow early mobilization, thus minimizing side effects such as joint stiffness and muscle wasting. While the anterolateral deltoid splitting approach using a locking plate is popularly adopted to enhance early osseous union and functional recovery in proximal humerus fractures, some studies reported inferior outcomes and potential construct failures in aging population and in complex fractures [19–22]. The impact of age on the outcome is generally of concern since surgical fixation is getting more popular in this fracture entity in an increasing aging population as well as in patients with severe fractures [23, 24]. In our series, we found no significant differences in functional outcomes and complication rates between the patients younger than and older than 60 years; both age groups had an almost equal number of patients with complex fracture patterns. Female gender was also considered as an independent risk factor for proximal humerus fractures owing to lower bone density and bone fragility [22]; however, there was no gender-specific difference in fracture healing and functional outcome in our series.

On reviewing postoperative radiographic analysis, it was found that the optimal correction of proper neck-shaft angles was critically important in maintaining realignment and avoiding varus collapse. This was a common complication in elderly patients and in patients with complex fractures [9]; it seemed to be a major contributor to satisfactory outcomes [22, 25]. A cut-off value of 105^o^ was associated with a significant difference in the functional scores and it could be considered as radiographic criteria for fracture reduction.

Nevertheless, common criticism about the deltoid splitting approach regarding potential axial nerve and deltoid muscle damage exists [10]. Based on anatomical studies, a safe area for lateral deltoid splitting was calculated from the acromial end as <5.2 cm by distance or as <0.19 in ratio to arm length [26, 27]. In our surgeries, we constantly limited the deltoid split to approximately 3 cm in length from the acromial end, and this resulted in no cases of iatrogenic axial nerve injury. Complication rate was comparable to other reports with the deltoid splitting approach for either plating or nailing humerus fractures [8, 9, 11, 28]. During fracture reduction and implant application, the deltoid split was used as a “moving window” by gently rotating the proximal humerus to expose the fracture site. The tension of the deltoid muscle around this moving window could be further released with arm abduction, thereby avoiding forceful retraction. It has been recommended that using a stepwise surgical procedure and a longer plate may avoid nerve damage and achieve reliable outcomes [8]. While the deltoid function was not quantified by any electrophysiological studies, none of the patients complained of sensation change or weakness around the deltoid muscle.

Surgical complications were experienced by as many as 38% of our patients, which was another important concern. A relatively high incidence of screw protrusion was found in both age groups. A previous study reported a radiographic complication rate of 36% in their patients, with a 43% rate of cutout in patients over 60 years [29]. In a review of 12 studies with locking plate fixation, the overall rate of complications was 49%, including screw perforation, malunion, and nonunion [30]. This study found that an overall complication rate of 18.2% was presumed to be due to the use of an indirect reduction maneuver and a minimally invasive technique. Since a limited surgical field and dissection may certainly jeopardize the proper placement of implants, it is crucially important to have meticulous intraoperative examination of reduction quality and an awareness of shortened screw length while maintaining secure fixation.

The main limitations of this study include the small cohort number and the heterogeneity of fracture patterns. Furthermore, deltoid muscle function was not quantified and compared using different surgical options. In addition, the lack of a randomized control may be critical. Finally, a follow-up study of osseous necrosis, preferably after a long interval, should be conducted.

## Conclusions

The deltoid splitting approach for proximal humeral fractures provided good functional results and early osseous union in both age groups. Better functional scores were obtained in male patients. A tendency to achieve a superior outcome was observed when the neck-shaft angles were well corrected. Implant-related complications, most frequently screw penetration, continue to be a common concern using locking plate fixation in the region.

## Abbreviations

LCP: Locking Compression Plate
MIPO: Minimally invasive plate osteosynthesis
